# The Standard Dictionary of Funk & Wagnalls

**Published:** 1895-08

**Authors:** 


### The Standard Dictionary of Funk & Wagnalls.

"Three eminent foreign scholars, Professors Skeat, Max Müller, and A. H. Sayce, have united in giving highest praise to the new American dictionary, published by Funk & Wagnalls. Professor Sayce, of Oxford University, is quoted as saying: 'The *Standard Dictionary* is truly magnificent, and worthy of the great continent which produced it. It is more than complete, and the amount of labor that has been bestowed upon it, and more especially upon the settlement of the pronunciation, must have been enormous. It is certain to supersede all other existing dictionaries of the English language.' "—*The New York Tribune*, July 9th, 1895.

